# Degradation of Patulin in Pear Juice and Apple Juice by Ascorbic Acid and the Combination of Ascorbic Acid and Ferrous Iron

**DOI:** 10.3390/toxins14110737

**Published:** 2022-10-28

**Authors:** Xiaoyan Wei, Mengyao Du, Sung-Yong Hong, Ae-Son Om

**Affiliations:** Department of Food and Nutrition, College of Human Ecology, Hanyang University, Seoul 04763, Korea

**Keywords:** apple juice, ascorbic acid, degradation, ferrous iron, patulin, pear juice

## Abstract

Patulin (PAT) is a toxic secondary metabolite produced by certain species of *Penicillium* sp. and *Aspergillus* sp. on apples and pears. In this study, we investigated the effects of ascorbic acid and the combination of ascorbic acid and ferrous iron on degradation of PAT in 100% pure pear juice and apple juice using high-performance liquid chromatography UV detector (HPLC-UVD). The addition of 2 different levels of ascorbic acid (143 or 286 μg/mL) into pear juice or apple juice containing 0.08 or 0.4 μg/mL of PAT showed 87.7–100% and 67.3–68.7% of PAT degradation rates, respectively, after 24 h incubation at 25 °C. Moreover, the addition of both ascorbic acid (143 or 286 μg/mL) and ferrous iron (0.033 or 0.11 μmol/mL) into pear juice or apple juice containing the same level of PAT exhibited higher PAT degradation rates (100 and 75–94%, respectively) than the addition of only ascorbic acid after 24 h incubation at 25 °C. Our data demonstrated that ascorbic acid plus ferrous iron as well as ascorbic acid were highly effective on degradation of PAT in pear juice and apple juice and that addition of both ascorbic acid and ferrous iron produced higher PAT degradation rates than addition of only ascorbic acid.

## 1. Introduction

Patulin (4-hydroxy-4H-furo[3,2c]pyran-2[6H]-one, PAT) is a mycotoxin produced by more than 60 species of fungi including over 30 genera common to fruit and vegetable-based products, such as apples, cherries, plums, apricots, peaches, nectarines, and pears and their products [1]. PAT contamination on apples or pears is usually associated with apple soft rot and blue mold rot commonly caused by *Penicillium expansum* [2]. PAT has been detected in pear at lower and less dangerous levels than those observed in apples [3]. PAT contamination mainly occurs during postharvest storage of the fruits. In particular, the PAT contamination in apple products poses a serious health risk to consumer, particularly children who consume increased levels of apple products during the 1st year of their life (6.4 g/kg BW/d) compared with adults (1 g/kg BW/d) [1,4]. Assessment of the health risks posed by PAT to humans suggests that PAT consumption can cause acute symptoms such as pulmonary congestion, edema, ulceration, intestinal hemorrhage and inflammation, epithelial cell degeneration, and gastrointestinal and kidney damage [1]. Chronic health risks of PAT consumption can include neurotoxic, immunotoxic, genotoxic, teratogenic, and carcinogenic effects [1,5,6]. PAT toxicity is believed to result from thiol group-related interactions [1]. PAT can cause cell damage by forming adducts with thiol-containing cellular components such as glutathione and cysteine-containing proteins and inducing intra- and intermolecular protein cross-links with the thiol group of cysteine or the side chains of lysine, histidine, and α-amino groups [7]. As a result, it can produce inhibition of protein and DNA syntheses and disruption of transcription and translation [1]. The health risks posed by patulin necessitate its control and removal from apple products. Therefore, the Codex Alimentarius Commission (CAC) has set a maximum permissible level (50 µg/L) for apple-based products [8]. Many regulation agencies including European Commission, the US Food and Drug Administration (FDA), and Korea Ministry of Food and Drug Safety (MFDS) began to place limitation on the PAT content in foods such as single-strength and reconstituted apple juice [9,10].

A number of studies to remove PAT in apple juice reported physical methods such as the use of activated carbon, chemical treatments such as the use of food-grade additives, and biological control methods such as microbial fermentation [1,11,12,13]. However, of the 3 control methods, the physical method negatively affected color, pH, and Brix of apple juice, and the biological method was limited to products that can be fermented [1,12]. Thus, one of the promising PAT detoxification methods for apple juice is utilization of food-grade additives such as vitamins including ascorbic acid, thiamine hydrochloride, pyridoxine hydrochloride, and calcium-D-pantothenate [14,15]. In particular, several previous studies have shown that ascorbic acid can decrease levels of PAT in aqueous solutions including apple juice [15,16,17,18]. One study showed that an aqueous solution with addition of ascorbic acid had 2.5-fold higher PAT degradation rate than that without ascorbic acid at acidic pH (pH 4) after 34-day incubation [15]. In another study, it was shown that cloudy apple juice containing 100 ng/g of PAT and 2,500 μg/mL of ascorbic acid produced much higher PAT degradation rates than that containing the same level of PAT without ascorbic acid after 3 or 6 days of incubation at 22 °C [18]. It has been also reported that ascorbic acid acts as a nucleophilic promoter for PAT degradation in clear or cloudy apple juice and juice-like aqueous solutions [15,16,17,18,19,20]. Besides, it was shown that ferrous iron can be used for metal-catalyzed oxidation of ascorbic acid to generate free radicals such as hydrolyl radicals or singlet oxygen, which can affect degradation of PAT [15]. Moreover, Drusch and co-workers showed that ferrous iron or a combination of hydrogen peroxide and ferrous iron can induce a rapid degradation of PAT in an aqueous solution [15]. Hence, in this study we investigated the PAT degradation rates in pear juice or apple juice by the addition of 2 different levels of ascorbic acid as well as the combination of the 2 different levels of ascorbic acid and 2 different levels of ferrous iron over 24 h incubation at 25 °C. In addition, we compared the PAT degradation rates in pear juice with those in apple juice under the same conditions.

## 2. Results

### 2.1. Validation of the Analytical Method Using High-Performance Liquid Chromatography-UV Detector (HPLC-UVD)

The analytical method using HPLC-UVD was validated for analytical parameters including linearity, accuracy, precision, and sensitivity. The linearity of a series of PAT concentrations in the analytical method was assessed by each standard curve using 5 levels of PAT standard solutions for pear juice or apple juice. The linearity of the calibration curves was determined by linear regression analysis. The curves for pear juice and apple juice showed *r*^2^ values of 0.995 and 0.998, respectively (Figure S1A,B). Therefore, we concluded that the standard curves were linear in the range of 0–2 μg/mL. 

The accuracy of the method was evaluated by the recovery of PAT extracted from pear juice or apple juice, which was fortified with known concentrations of PAT standard solutions (0.2, 0.5, and 1 μg/mL), and the recoveries were calculated by the following equation.

$$Recovery\;\left(\%\right)=\frac{Patulin\;concentration\;equivalent\;to\;the\;peak\;area\;measured\;from\;the\;spiked\;sample\times 100}{\begin{array}{c} Patulin\;concentration\;used\;for\;spiking\;the\;sample \end{array}}$$

The recovery rates for PAT in pear juice were in the range of 75–81%, while those in apple juice were in the range of 86–95% (Table S1). The recovery rates in all of the samples were higher than 75%, which is in agreement with the recovery rates (75–105%) recommended by Official Journal of the European Union [21]. Thus, it was concluded that the analytical method had good recoveries from pear juice and apple juice.

The precision of the method was assessed by repeatability (within-day precision). The relative standard deviation (*RSDr*) obtained from PAT in pear juice was in the range of 7.5–8.5%, while that in apple juice was in the range of 6.3–13.1% (Table S1). Those values were consistent with the reference level (≤15%) recommended by Official Journal of the European Union [21]. Hence, we concluded that the analytical method showed good precision in determination of PAT in pear juice or apple juice.

**Figure 1:**
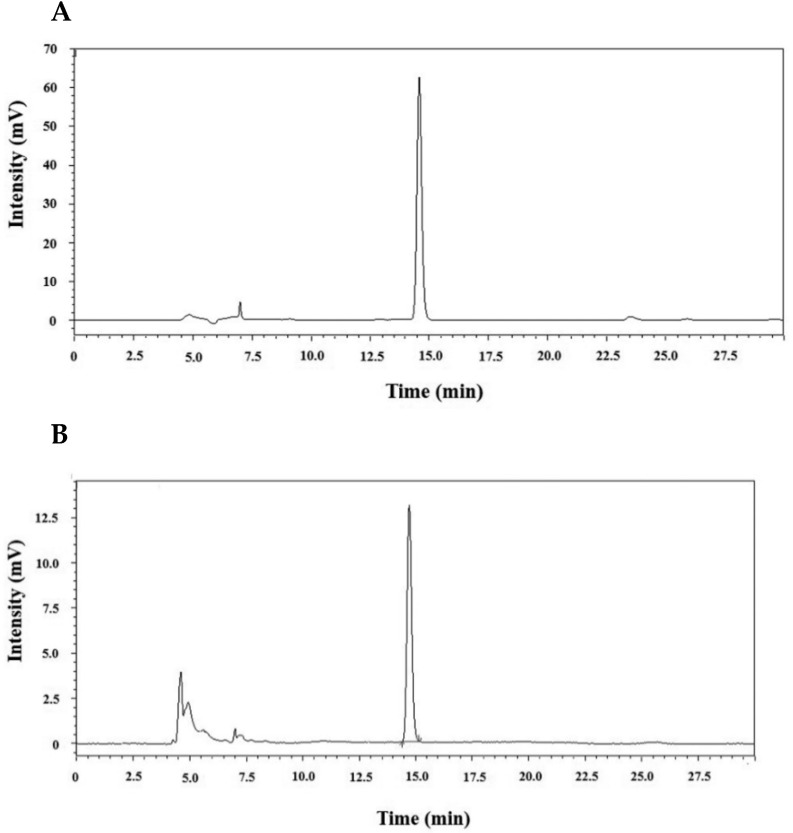

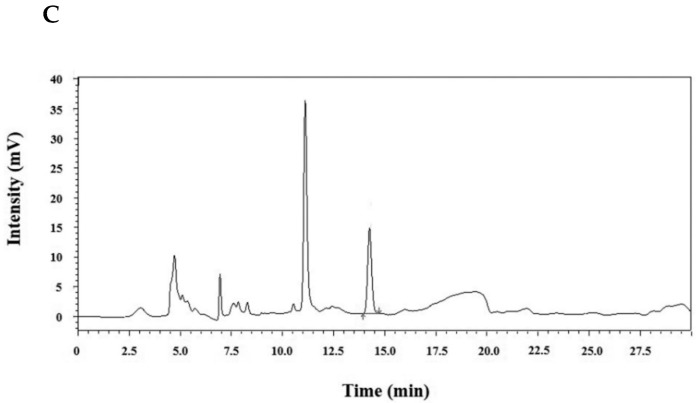
Chromatograms of PAT using HPLC-UVD. Chromatograms of (**A**) PAT standard solution (1 μg/mL) and of PAT extracted from (**B**) pear juice and (**C**) apple juice. The retention time of the PAT peak was 14.7 min.

The sensitivity of the analytical method using HPLC-UVD was determined by a limit of detection (LOD) and limit of quantification (LOQ). The LOD and LOQ for PAT in pear juice were 0.015 and 0.045 μg/mL, while those in apple juice were 0.01 and 0.03 μg/mL, respectively. They were as low as those for detection of trace amounts of PAT. It indicates that the method was highly sensitive for determination of PAT in pear juice and apple juice. Figure 1 shows the representative chromatograms of PAT in standard solution and extracted from pear juice or apple juice. 

### 2.2. Effects of Ascorbic Acid on Degradation of PAT in Pear Juice or Apple Juice

**Figure 2:**
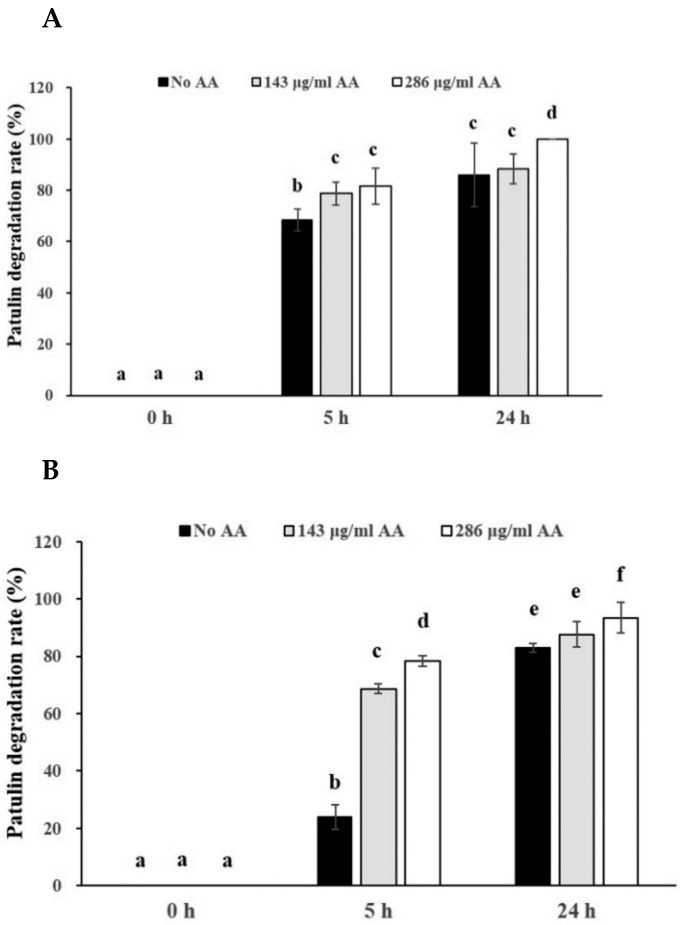
Effects of 2 different levels of ascorbic acid on degradation of 2 different levels of PAT in pear juice for 24 h incubation at 25 °C. The pear juice containing (**A**) 0.08 or (**B**) 0.4 μg/mL of PAT was incubated in the presence of 143 or 286 μg/mL of ascorbic acid for 5 or 24 h at 25 °C. The PAT was then extracted from the samples. The levels of PAT were measured in triplicate. The values are expressed as the mean ± standard error. Different letters in the same group indicate statistically significant differences (*p* < 0.05).

In order to investigate the effects of ascorbic acid on degradation of PAT in pear juice or apple juice, pear juice or apple juice containing two levels of PAT (0.08 or 0.4 μg/mL) was incubated with two levels of ascorbic acid (143 or 286 μg/mL) for 0, 5, and 24 h at 25 °C. The PAT degradation rates in pear juice containing the low level of PAT (0.08 μg/mL) were gradually increased regardless of the levels of ascorbic acid as incubation time became longer (Figure 2). The degradation rates in pear juice containing 0.08 μg/mL of PAT after 24 h incubation (88.3% for 143 μg/mL of ascorbic acid, 100% for 286 μg/mL of ascorbic acid) were increased relative to those after 5 h incubation (78.7% for 143 μg/mL of ascorbic acid, 88.3% for 286 μg/mL of ascorbic acid) (Figure 2A). Additionally, the addition of the high level of ascorbic acid into pear juice had an increased trend of the PAT degradation rate, compared to pear juice containing the low level of ascorbic acid at the same incubation time. In particular, the PAT degradation rates between 2 pear juice samples containing different levels of ascorbic acid showed statistically significant difference (*p* < 0.05) at 24 h incubation (Figure 2A).

The PAT degradation rates in pear juice containing the high level of PAT (0.4 μg/mL) showed a similar pattern to those in pear juice containing the low level of PAT (0.08 μg/mL) (Figure 2B). After 24 h incubation, the degradation rates in pear juice (87.7% for 143 μg/mL of ascorbic acid, 93.5% for 286 μg/mL of ascorbic acid) were increased, compared to those at 5 h incubation (68.7% for 143 μg/mL of ascorbic acid, 78.3% for 286 μg/mL of ascorbic acid) after the addition of the same level of ascorbic acid. In addition, pear juice in the presence of the high level of ascorbic acid showed an increased tendency of the PAT degradation rates relative to that in the presence of the low level of ascorbic acid at the same incubation time. The PAT degradation rates between 2 pear juice samples containing different levels of ascorbic acid showed statistically significant difference (*p* < 0.05) at 5 and 24 h incubation (Figure 2B). However, when the PAT degradation rates were compared between 2 pear juice samples containing the different levels of PAT (0.08 or 0.4 μg/mL) at the same incubation time after the addition of the same levels of ascorbic acid, there were no statistically significant difference between them (Figure 2A,B).

**Figure 3:**
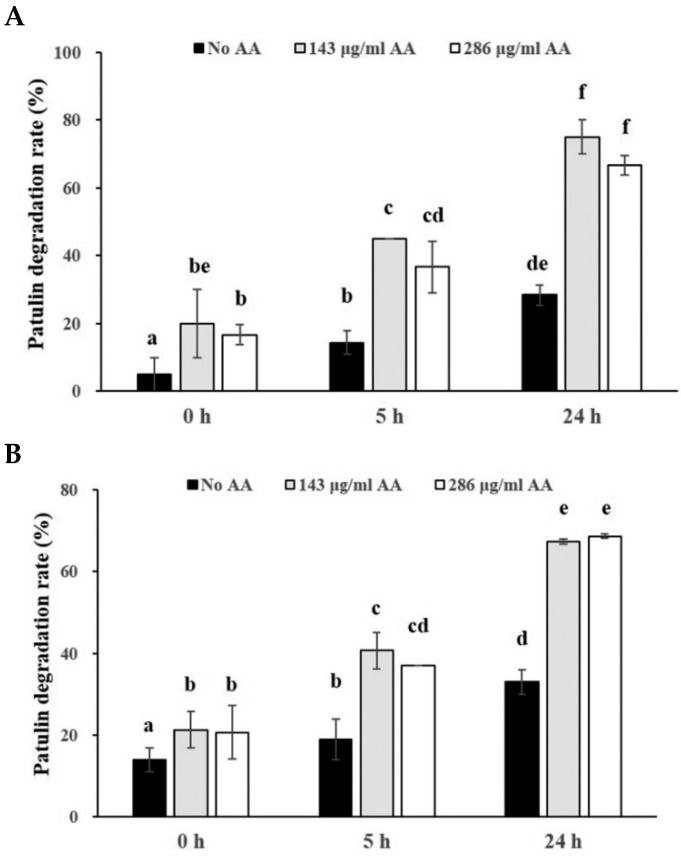
Effects of 2 different levels of ascorbic acid on degradation of 2 different levels of PAT in apple juice for 24 h incubation at 25 °C. The apple juice containing (A) 0.08 or (B) 0.4 μg/mL of PAT was incubated in the presence of 143 or 286 μg/mL of ascorbic acid for 5 or 24 h at 25 °C. The PAT was then extracted from the samples. The levels of PAT were measured in triplicate. The values are expressed as the mean ± standard error. Different letters in the same group indicate statistically significant differences (*p* < 0.05).

Similarly, the PAT degradation rates in apple juice containing the low level (0.08 μg/mL) of PAT were also gradually increased as incubation time passes by (Figure 3A). The degradation rates in apple juice containing 0.08 μg/mL of PAT after 24 h incubation (75% for 143 μg/mL of ascorbic acid, 66.7% for 286 μg/mL of ascorbic acid) were increased, compared to those after 5 h incubation (45% for 143 μg/mL of ascorbic acid, 36.7% for 286 μg/mL of ascorbic acid) (Figure 3A). However, apple juice, to which the high level of ascorbic acid was added, did not increase the PAT degradation rates at the same incubation time, relative to that in the presence of the low level of ascorbic acid (Figure 3A). These results were different from those in pear juice, in which the addition of the high level of ascorbic acid increased the PAT degradation rates. Interestingly, apple juice samples at 0 h incubation also showed approximately 15–20% of PAT degradation rates regardless of the levels of ascorbic acid.

The PAT degradation rates in apple juice containing the high level of PAT (0.4 μg/mL) had a similar pattern to those in apple juice containing the low level of PAT (0.08 μg/mL) (Figure 3B). The degradation rates in apple juice after 24 h incubation (67.3% for 143 μg/mL of ascorbic acid, 68.7% for 286 μg/mL of ascorbic acid) were increased relative to those after 5 h incubation (40.7 for 143 μg/mL of ascorbic acid, 37% for 286 μg/mL of ascorbic acid). However, the addition of the high level of ascorbic acid into apple juice samples containing the high level of PAT did not increase PAT degradation rates at the same incubation time, compared to the addition of the low level of ascorbic acid (Figure 3B). These data were similar to those from apple juice containing the low level of PAT (Figure 3A). In addition, when the PAT degradation rates were compared between 2 apple juice samples containing the different levels of PAT (0.08 or 0.4 μg/mL) at the same incubation time after the addition of the same levels of ascorbic acid, no statistically significant difference between them was observed (Figure 3A,B). Additionally, apple juice samples containing the high level of PAT at 0 h incubation showed approximately 20% of PAT degradation rates. The PAT degradation rates in apple juice containing either the low or high level of PAT at 0 h incubation were different from those in pear juice containing the same level of PAT at 0 h incubation described above, in which the rates were 0% (Figure 2A,B).

### 2.3. Effects of the Combination of Ascorbic Acid and Ferrous Iron on Degradation of PAT in Pear Juice or Apple Juice

**Figure 4:**
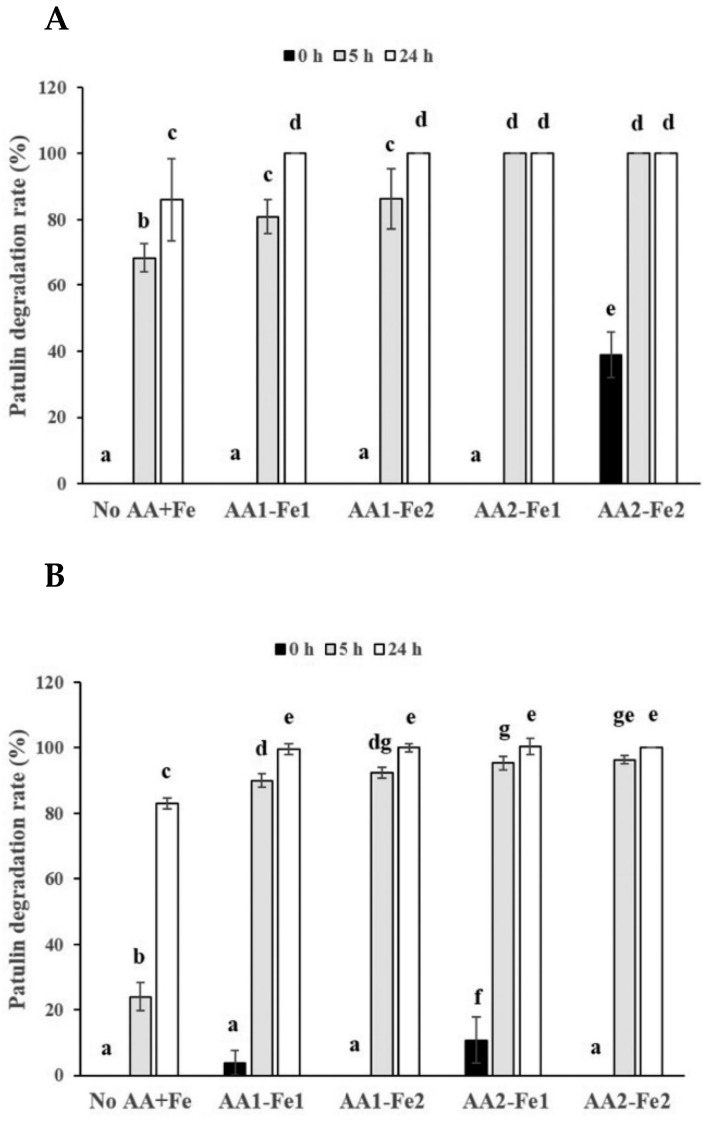
Effects of the combination of 2 different levels of ascorbic acid and ferrous iron on degradation of 2 different levels of PAT in pear juice for 24 h incubation at 25 °C. The pear juice containing (**A**) 0.08 or (**B**) 0.4 μg/mL of PAT was incubated in the presence of a combination of 143 or 286 μg/mL of ascorbic acid (AA1 and AA2, respectively) and 0.033 or 0.11 μmol/mL of ferrous iron (Fe1 and Fe2, respectively) for 5 or 24 h at 25 °C. The PAT was then extracted from the samples. The levels of PAT were measured in triplicate. The values are expressed as the mean ± standard error. Different letters in the same group indicate statistically significant differences (*p* < 0.05).

In order to investigate the effects of the combination of ascorbic acid and ferrous iron on degradation of PAT in pear juice or apple juice, pear juice or apple juice containing two levels of PAT (0.08 or 0.4 μg/mL) was incubated with a combination of two levels of ascorbic acid (143 or 286 μg/mL) and two levels of ferrous iron (0.033 or 0.11 μmol/mL) for 0, 5, and 24 h at 25 °C. As incubation time became longer, the PAT degradation rates in pear juice containing the low level of PAT (0.08 μg/mL) were gradually increased when the low level of ascorbic acid (143 μg/mL) were added regardless of the level of ferrous iron (Figure 4A). The degradation rates in pear juice containing 0.08 μg/mL of PAT at 24 h incubation (100%) after the addition of the low level of ascorbic acid were increased relative to those after 5 h incubation (80.8–86.3%) (Figure 4A). However, the degradation rates in pear juice containing the low level of PAT in the presence of the high level of ascorbic acid (286 μg/mL) regardless of the level of ferrous iron did not show any statistically significant difference between pear juice samples after 5 and 24 h incubation (both 100%). Additionally, after 24 h incubation, the degradation rates in pear juice containing the low level of PAT were 100% under any combination of the level of ascorbic acid and ferrous iron (Figure 4A). Besides, the addition of the high level of ferrous iron into pear juice containing the low level of PAT did not increase the PAT degradation rates at the same incubation time when either low or high level of ascorbic acid was added, compared to the addition of the low level of ferrous iron (Figure 4A). However, the addition of the high level of ascorbic acid into pear juice showed an increased trend of the PAT degradation rates after 5 h incubation regardless of the level of ferrous iron, compared to the addition of the low level of ascorbic acid (Figure 4A). These results are similar to those from addition of only 2 different levels of ascorbic acid in pear juice as described above (Figure 2A).

The PAT degradation rates in pear juice containing the high level of PAT (0.4 μg/mL) showed a similar pattern to those in pear juice containing the low level of PAT (0.08 μg/mL) (Figure 4B). The PAT degradation rates in pear juice containing the high level of PAT (0.4 μg/mL) showed a gradually increased tendency regardless of the level of ascorbic acid and ferrous iron as incubation time became longer (Figure 4B). The degradation rates in pear juice containing 0.4 μg/mL of PAT after 24 h incubation (99.6% for 143 μg/mL of ascorbic acid and 0.033 μmol/mL of ferrous iron, 99.9% for 143 μg/mL of ascorbic acid and 0.11 μmol/mL of ferrous iron) had an increased tendency relative to those after 5 h incubation (90% for 143 μg/mL of ascorbic acid and 0.033 μmol/mL of ferrous iron, 92.3% for 143 μg/mL of ascorbic acid and 0.11 μmol/mL of ferrous iron) (Figure 4B). Similarly, the degradation rates in pear juice containing the high level of PAT in the presence of the high level of ascorbic acid (286 μg/mL) regardless of the level of ferrous iron also had an increased trend as incubation time became longer (95.3% at 5 h vs. 100% at 24 h for 286 μg/mL of ascorbic acid and 0.033 μmol/mL of ferrous iron, 96.3% at 5% vs. 100% at 24 h for 286 μg/mL of ascorbic acid and 0.11 μmol/mL of ferrous iron) (Figure 4B). Furthermore, the addition of the high level of ferrous iron into pear juice did not statistically significantly increase the PAT degradation rates at the same incubation time after either low or high level of ascorbic acid was added, compared to the low level of ferrous iron added (Figure 4B). Additionally, the addition of the high level of ascorbic acid into pear juice containing the high level of PAT showed an increased trend of the PAT degradation rates after 5 h incubation regardless of the level of ferrous iron, compared to the addition of the low level of ascorbic acid (Figure 4B), which are similar results to those in pear juice containing the low level of PAT (Figure 4A) and to those from addition of only 2 different levels of ascorbic acid in pear juice (Figure 2A) as described above. Interestingly, the degradation rates in pear juice containing 0.4 μg/mL of PAT (90.0–92.3%) were increased relative to those in pear juice containing 0.08 μg/mL of PAT (80.8–86.3%) at 5 h incubation after addition of the low level of ascorbic acid (143 μg/mL) regardless of the level of ferrous iron (Figure 4A,B).

**Figure 5:**
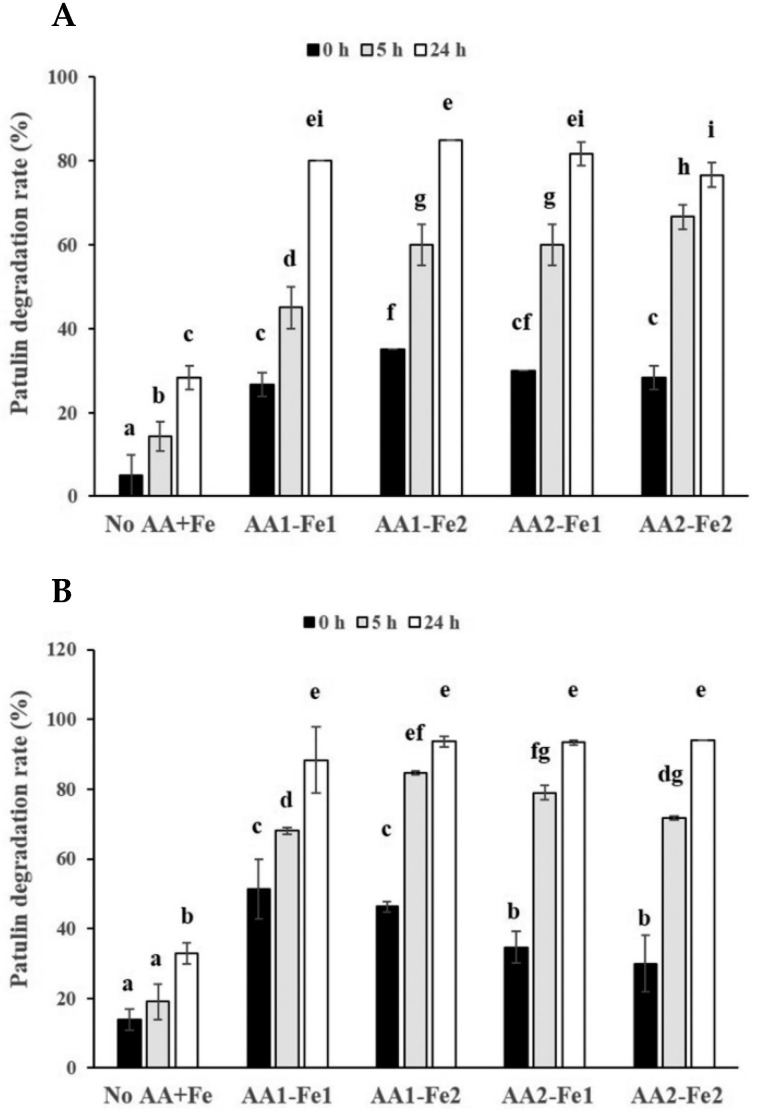
Effects of the combination of 2 different levels of ascorbic acid and ferrous iron on degradation of 2 different levels of PAT in apple juice for 24 h incubation at 25 °C. The apple juice containing (**A**) 0.08 or (**B**) 0.4 μg/mL of PAT was incubated in the presence of a combination of 143 or 286 μg/mL of ascorbic acid (AA1 and AA2, respectively) and 0.033 or 0.11 μmol/mL of ferrous iron (Fe1 and Fe2, respectively) for 5 or 24 h at 25 °C. The PAT was then extracted from the samples. The levels of PAT were measured in triplicate. The values are expressed as the mean ± standard error. Different letters in the same group indicate statistically significant differences (*p* < 0.05).

Similarly, the PAT degradation rates in apple juice containing the low level of PAT (0.08 μg/mL) were also gradually increased when it was incubated with a combination of two levels of ascorbic acid (143 or 286 μg/mL) and two levels of ferrous iron (0.033 or 0.11 μmol/mL) as the incubation time became longer (Figure 5A). The degradation rates in apple juice containing 0.08 μg/mL of PAT after 24 h incubation (76.7–85.0%) were increased relative to those after 5 h incubation (45.0–66.7%) regardless of the level of ferrous iron. Furthermore, the addition of the high level of ferrous iron into apple juice containing the low level of PAT statistically significantly increased the PAT degradation rates at 5 h incubation after either low or high level of ascorbic acid was added, compared to the low level of ferrous iron added (*p* < 0.05) (Figure 5A), which are different from results in pear juice containing PAT as described above (Figure 4). Additionally, the addition of the high level of ascorbic acid into apple juice containing the low level of PAT did not increase the PAT degradation rates at the same incubation time, compared to the addition of the low level of ascorbic acid (Figure 5A). These results were slightly different from those in pear juice containing the same level of PAT, in which addition of the high level of ascorbic acid increased the PAT degradation rates at 5 h incubation (Figure 4A). Interestingly, apple juice samples containing the low level of PAT at 0 h incubation also showed approximately 30% PAT degradation rates regardless of the level of ascorbic acid or ferrous iron.

The PAT degradation rates in apple juice containing the high level of PAT (0.4 μg/mL) had a similar pattern to those in apple juice containing the low level of PAT (0.08 μg/mL) (Figure 5B). The PAT degradation rates in apple juice containing the high level of PAT (0.4 μg/mL) after 24 h incubation (88.3–94.0%) were increased relative to those after 5 h incubation (68.0–84.7%) regardless of the level of ascorbic acid or ferrous iron. In addition, similar to the results from apple juice containing the low level of PAT, the addition of the high level of ascorbic acid into apple juice containing the high level of PAT did not statistically significantly increase the PAT degradation rates at the same incubation time, compared to the addition of the low level of ascorbic acid (Figure 5B). However, apple juice containing the high level of PAT in the presence of the high level of ferrous iron statistically significantly increased the PAT degradation rates at 5 h incubation after low level of ascorbic acid was added, compared to that in the presence of the low level of ferrous iron added (*p* < 0.05), which are similar to the results from apple juice containing the low level of PAT as described above. Additionally, the degradation rates in apple juice containing 0.4 μg/mL of PAT (68.0–84.7% and 88.3–94.0% for 5 and 24 h incubation, respectively) were increased relative to those in apple juice containing 0.08 μg/mL of PAT (45.0–66.7% and 76.7–85.0% for 5 and 24 h incubation, respectively) at the same incubation time after addition of the same level of ascorbic acid or ferrous iron (Figure 5A,B), which are similar to the data from apple juice samples containing the low level of PAT. In addition, the apple juice containing the high level of PAT also showed approximately 30% of PAT degradation rates at 0 h incubation regardless of the level of ascorbic acid or ferrous iron.

### 2.4. Confirmation of PAT in Pear Juice or Apple Juice by Liquid Chromatography-Tandem Mass Spectrometry (LC/MS/MS)

**Figure 6:**
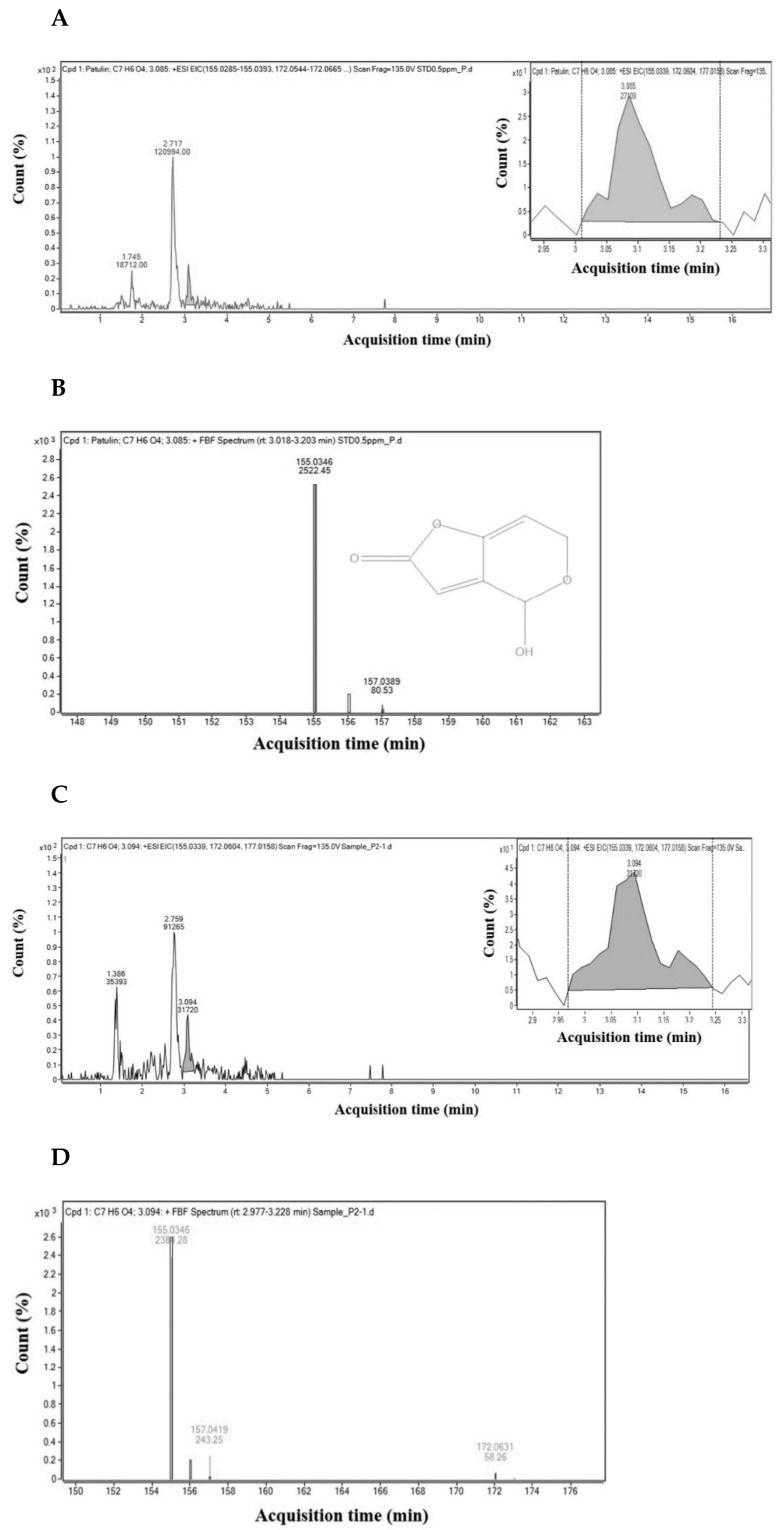
Extracted ion chromatogram and MS spectrum of PAT. (**A**) Extracted ion chromatogram. (EIC) and (**B**) MS spectrum obtained by the Find by Formula algorithm for PAT in a PAT standard solution, and (**C**) EIC and (**D**) MS spectrum for PAT in apple juice. (Inset) The retention time (3.083 min) of the PAT peak in EIC.

The PAT in pear juice or apple juice, which was detected by HPLC-UVD, was further confirmed by LC/MS/MS. The PAT in a standard solution (1 μg/mL) and apple juice were eluted at 3.085 and 3.094 min, respectively (Figure 6A,C). The mass-to-charge (*m*/*z*) ratio of the most abundant product ion which results from collision-induced dissociation (CID) of PAT in the standard solution or apple juice was 155.03 (Figure 6B,D). Additionally, the *m*/*z* ratio of the most abundant product ion of PAT in the standard solution or pear juice was 155.03 (data not shown). Thus, these data confirm the identity of PAT detected in apple juice or pear juice samples by HPLC-UVD after 5 or 24 h incubation.

## 3. Discussion

This study aimed to investigate the effects of either 2 different levels of ascorbic acid or a combination of 2 different levels of ascorbic acid and 2 different levels of ferrous iron on degradation rates of PAT in pear juice or apple juice after 5 or 24 h incubation at 25 °C. In the present study, we first validated the analytical method for determination of PAT by HPLC-UVD using the parameters such as linearity, accuracy, precision, and sensitivity. The analytical method showed good linearity, sensitivity, specificity, and accuracy in the determination of PAT by HPLC-UVD. The method established in our study was validated for 2 different matrices at levels as low as those of PAT in apple juice regulated by many regulation agencies. 

Next, we analyzed PAT degradation rates in pear juice or apple juice containing 2 different levels of PAT after addition of 2 different levels of ascorbic acid over 24 h incubation at 25 °C. The addition of both levels of ascorbic acid into pear juice or apple juice containing PAT accelerated the PAT degradation rates. Furthermore, the level of PAT in pear juice after 24 h incubation (less than 28 ng/mL in the juice containing 0.4 μg/mL of PAT), which are equivalent to more than 93% PAT degradation rates, were below the maximum allowable legal limit of PAT (50 ng/mL) in apple juice set by the MFDS or US FDA when the limit of PAT in apple juice is used for comparison since the legal limit of PAT in pear juice is not yet set worldwide as well as in Korea (Figure 2A,B). After incubation with ascorbic acid, the remaining PAT in pear juice or apple juice detected by HPLC-UVD was confirmed by LC/MS/MS.

It has been reported that high stability of PAT was observed in the range of pH 3.5–5.5 of aqueous solutions [15,17,22]. Drusch and co-workers (2007) showed that 69–73% of the initial PAT level (2 μg/mL) was still present in pH 2.5–5.5 of aqueous buffer systems after 30-day incubation [15]. Another study showed that the PAT degradation rate in an aqueous buffer solution (pH 7.5) containing 5000 ng/mL of PAT was approximately 45% at 25 °C after 24 h incubation with addition of 20,000 μg/mL of sodium ascorbate, while that in another buffer solution (pH 3.5) containing the same level of PAT was approximately 20% at the same temperature after the same incubation time with addition of 30,000 μg/mL of a mixture of ascorbic acid and sodium ascorbate [17]. Since pH of the apple juice (pH 4.5) in our study was similar to a typical pH of apple juice (pH 4), it is possible that PAT in apple juice that we used was relatively high stable without addition of ascorbic acid as described above. The PAT degradation rates in the present study were 5–33% in apple juice containing PAT (0.08 or 0.4 μg/mL) without addition of ascorbic acid (Figure 3 and Figure 5). It is in agreement with the previous results, in which the degradation rate was 14% in pH 4 of an aqueous solution containing 2 μg/mL of PAT after 9-day incubation without addition of ascorbic acid, while those were approximately 30% in pH 2.5–5.5 of aqueous buffer systems containing 2 μg/mL of PAT after 30day incubation without addition of ascorbic acid [15].

Besides, in the current study the addition of ascorbic acid (143 or 286 μg/mL) into apple juice containing PAT (0.08 or 0.4 μg/mL) also increased the PAT degradation rates by 2–2.5 fold after 5 or 24 h incubation, compared to no addition of ascorbic acid (36.7–75.0% for addition of ascorbic acid, 14.3–33.0% for no addition of ascorbic acid) (Figure 3A,B). These results are consistent with those in previous studies [15,16,17,18,20]. Drusch and co-workers (2007) described that addition of ascorbic (482 μg/mL) increased the PAT degradation rate to 59% by approximately 4 fold in pH 4 of an aqueous solution containing 2 μg/mL of PAT after 9-day incubation, compared to no addition of ascorbic acid (14%) [15]. However, although pH of pear juice used in the current study was 3.7, which is similar to that in apple juice (pH 4.5), the addition of ascorbic acid into pear juice containing the same level of PAT with those in the apple juice increased the PAT degradation rates by only approximately 1.2 fold after 5 or 24 h incubation, compared to no addition of ascorbic acid (68.7–100% for addition of ascorbic acid, 68.5–85.9% for no addition of ascorbic acid except the rate at 5 h incubation) (Figure 2A,B). In addition, the overall PAT degradation rates were higher in pear juice than in apple juice (Figure 2 and Figure 3). These data in our study were in line with those in some previous studies, in which the authors demonstrated the greater stability of PAT in apple juice than in aqueous buffer systems, the pH of which was similar to that of the former [17,20]. A previous study reported that the PAT degradation rate in apple juice was slower than those in aqueous buffer systems at 25 °C after 4-day incubation with addition of ascorbic acid (35% for apple juice with 5% of ascorbic acid mixture, 60% for pH 3.5 of buffer solutions with 3% of ascorbic acid mixture) [17]. The similar result was shown in another study from Chile, in which the PAT degradation rate in apple juice (pH 3.5) containing 450 μg/mL of PAT was lower than that in the same pH of an aqueous solution containing of the same level of PAT (25 and 54% at 0 h incubation after addition of 100 μg/mL of ascorbic acid, respectively), whereas the degradation rate in the apple juice containing 450 μg/mL of PAT was approximately 50% at 72 h incubation after addition of 100 μg/mL of ascorbic acid [20]. These results might have come from protective and antioxidative effects of polyphenols in apple juice against oxidation of ascorbic acid by O_2_ because it was reported that addition of ascorbic acid into apple juice inhibits polyphenol oxidase activity, resulting in preventing the degradation of polyphenol in apple juice [23]. On the other hand, some researchers postulated a possible mechanism of PAT degradation accelerated by ascorbic acid [15,17]. They presumed metal-catalyzed oxidation of ascorbic acid to dehydroascorbic acid, which results in generation of reactive free radicals such as hydroxyl radicals or singlet oxygen that can attack the conjugated double bonds of patulin, causing the opening of the lactone ring [15,17]. Other studies proposed another mechanism, in which electrophilic properties of PAT may make a suitable target for nucleophilic attack by ascorbic acid [19,24,25,26]. It seems that both or either degradation mechanism is possible depending on the amount of oxygen in samples. Thus, as we described above, higher levels of antioxidative capacity due to polyphenol in apple juice than those in pear juice might have prevented oxidation of ascorbic acid in apple juice. Another possible reason might be the different initial contents of ascorbic acid in pear juice and apple juice. One previous study reported that 1 L of pure apple juice contains 3.9 mg of ascorbic acid, while 1 L of pear juice contains 30.6 mg of ascorbic acid [23,27]. Since pear juice contains approximately 7-fold higher amount of ascorbic acid than apple juice, it seems that in this study the PAT degradation rates in all pear juice samples as well as control samples without addition of ascorbic acid were higher than those in apple juice samples.

Additionally, a previous study described by Fremy et al., (1995) reported that addition of 30,000 μg/mL of ascorbic acid into an aqueous solution containing 0.1 μg/mL of PAT showed 5 and 36% of PAT degradation rates after 3 and 44 h incubation at room temperature, respectively [16]. In our study, the PAT degradation rates in apple juice or pear juice containing the same level of PAT were also gradually increased as the incubation time became longer after addition of the same level of ascorbic acid as described above (Figure 2 and Figure 3). Interestingly, one study showed that the PAT degradation rates in pH 4 of an aqueous solution containing 2 μg/mL of PAT were gradually increased even without addition of ascorbic acid as the incubation time became longer (14% after 9 days, 32% after 34 days) [15]. Another study also showed that PAT degradation rates in cloudy apple juice containing 100 ng/mL of PAT without addition of ascorbic acid were 45 and 55% after 0- and 3-day incubation at 22 °C, respectively [18]. In the present study, we also observed the gradually increased PAT degradation rates in apple juice or pear juice containing PAT (0.08 or 0.4 μg/mL) without addition of ascorbic acid as the incubation time passes by (Figure 2 and Figure 3).

Several studies about PAT degradation in the presence of ascorbic acid showed that the degradation rates were very different depending on the initial levels of PAT and ascorbic acid as well as incubation time [15,17,28]. Valletrisco and collaborators (1991) reported that an increase in the level of PAT in apple juice to 800 ng/mL from 500 ng/mL accelerated the PAT degradation rates in the presence of the same level of ascorbic acid after 8-day incubation (from 65% to 78% of the degradation rate for 150 μg/mL of ascorbic acid, from 50% to 70% of the degradation rate for 300 μg/mL of ascorbic acid) [28]. However, in our study, the PAT degradation rates in apple juice or pear juice containing the higher level of PAT (400 ng/mL) were not increased, compared to those in apple juice or pear juice containing the low level of PAT (80 ng/mL) at 5 or 24 h incubation after addition of the same level of ascorbic acid (Figure 2 and Figure 3). This discrepancy may have resulted from the shorter incubation time in this study than in the previous study.

Valletrisco and collaborators (1991) also showed that an increase in the level of ascorbic acid from 150 μg/mL to 300 μg/mL did not affect the PAT degradation rates in apple juice containing the same level of PAT after 8 days (65 and 50% of degradation rates for 500 ng/mL of PAT, 78 and 70% of degradation rates for 800 ng/mL of PAT, respectively) [28]. Another study reported that addition of 2 different levels of ascorbic acid (2500 or 40,000 μg/mL) produced similar degradation rates (72 and 73%, respectively) in cloudy apple juice containing 100 ng/mL of PAT after 3-day incubation at 22 °C [18]. These data are consistent with our results, in which the PAT degradation rates in apple juice containing the same level of PAT were not promoted by increasing the level of ascorbic acid from 143 μg/mL to 286 μg/mL (45.0 and 36.7% of degradation rates at 5 h incubation, and 75.0 and 66.7% at 24 h incubation, respectively, for 80 ng/mL of PAT, 40.7 and 37.0% of degradation rates at 5 h incubation, and 67.3 and 68.7% at 24 h incubation, respectively, for 400 ng/mL of PAT) (Figure 3A,B). However, the addition of the higher level of ascorbic acid into pear juice showed an increased tendency of the PAT degradation rates compared to the low level of ascorbic acid, which is a slightly different pattern to those in apple juice (Figure 2A,B). The possible reason might be the difference between the initial contents of ascorbic acid in pear juice and apple juice.

On the other hand, in a previous study, addition of 2 levels of ascorbic acid (2500 or 40,000 μg/mL) into cloudy apple juice containing 100 ng/ml of PAT showed 47 and 57% of PAT degradation rates, respectively, after 0-day incubation [18]. However, in the current study, addition of 2 levels of ascorbic acid (143 or 286 μg/mL) into apple juice or pear juice containing the same level of PAT (0.08 or 0.4 μg/mL) did not show any difference between them after 0 h incubation (Figure 2 and Figure 3). This discrepancy may have come from the smaller difference between the 2 levels of ascorbic acid added into apple juice or pear juice in our study than in the previous study. It suggests that the amount of ascorbic acid needed for PAT degradation is linked to the initial level of PAT in samples.

One previous study showed that rapid PAT degradation was induced by generation of free radicals through addition of hydrogen peroxide and ferrous iron into aqueous buffer solutions (pH 4) containing 1.2 μg/mL of patulin [15]. In their study, the addition of both hydrogen peroxide (14.7 μmol/mL) and ferrous iron (1.47 μmol/mL) increased the PAT degradation rate (96%) by 19 fold after 24 h incubation relative to the control without addition of both (5%). Furthermore, addition of ferrous iron (1.47 μmol/mL) alone also promoted the PAT degradation rate (27%) by approximately 5 fold after 24 h incubation compared to the control without addition of ferrous iron (5%). Therefore, in order to evaluate the effects of addition of both ascorbic acid and ferrous iron on PAT degradation rates, we added ferrous iron (0.033 or 0.11 μmol/mL) together with ascorbic acid (143 or 286 μmol/mL) into pear juice or apple juice containing PAT (0.08 or 0.4 μg/mL). The PAT degradation rates in pear juice or apple juice after addition of both ascorbic acid and ferrous iron were higher than those after addition of only ascorbic acid (68.7–78.3% for ascorbic acid and 90.0–96.3% for both ferrous iron and ascorbic acid after 5 h incubation in pear juice containing 0.4 μg/mL of PAT, 37.0–40.7% for ascorbic acid and 68.0–84.7% for both ferrous iron and ascorbic acid after 5 h incubation in apple juice containing 0.4 μg/mL of PAT) (Figure 2B and Figure 4B, Figure 3B and Figure 5B). It suggests that ferrous iron promoted oxidation of ascorbic acid in pear juice or apple juice, leading to higher PAT degradation rates. We speculate that the first one of the 2 mechanisms (free radical generation by metal-catalyzed oxidation of ascorbic acid to dehydroascorbic acid), which were described above, was predominant in the presence of both ascorbic acid and ferrous iron. The previous study described that in the first mechanism, a reaction of ascorbic acid with metal ions (ferrous iron in this study) produces reactive free radicals such as hydroxyl radicals or singlet oxygen that can attack the conjugated double bonds of patulin [17]. Another study, which supported the first mechanism, demonstrated that the level of dehydroascorbic acid (an oxidized form of ascorbic acid) was increased by addition of ascorbic acid (2500 μg/mL) into an aqueous solution (pH 4) containing 100 ng/ml of PAT as incubation time became longer to 24 days [18]. Moreover, in their study, they showed that treatment of cloudy apple juice containing PAT with ascorbic acid (2500 μg/mL) at 22 °C resulted in the generation of PAT degradation products less toxic than PAT, such as ascladiol-(E/Z) (ASC-E/Z) [18]. It is known that the ASC-E is the direct precursor of PAT in the biosynthetic pathway in PAT-producing fungi [29,30,31] and a predominant breakdown product of PAT from microorganisms such as *Saccharomyces cerevisiae* [32]. In addition, ASC-Z was detected as one of the final degradation products from *Sporobolomyces* sp. [33] and was a non-enzymatic transformation product of ASC-E catalyzed by sulfhydryl compounds such as cysteine or glutathione [34].

Moreover, our study showed that addition of the high level of ferrous iron (0.11 μmol/mL) did not increase the PAT degradation rates in pear juice at the same incubation time compared to addition of the low level of ferrous iron (0.033 μmol/mL), whereas the former increased the degradation rates in apple juice at 5 h incubation compared to the latter (Figure 4 and Figure 5). The possible reason for pear juice may be that addition of the 2 different levels of ferrous iron did not produce much different PAT degradation rates due to the high initial content of ascorbic acid in pear juice. However, the addition of the 2 different levels of ferrous iron into apple juice, which naturally contains the low level of ascorbic acid, may have produced significantly different PAT degradation rates.

Additionally, in the current study, the PAT degradation rates in apple juice or pear juice containing the higher level of PAT (400 ng/mL) were increased, compared to those in apple juice or pear juice containing the low level of PAT (80 ng/mL) at 5 or 24 h incubation after addition of the same levels of ascorbic acid or ferrous iron (Figure 4 and Figure 5), which was in agreement with the results by Valletrisco and co-workers (1991) as described above. These results suggest that the addition of both ferrous iron and ascorbic acid accelerated the PAT degradation rates more in apple juice or pear juice containing the higher level of PAT than those in apple juice or pear juice containing the low level of PAT.

In addition, in this study, the PAT degradation rates in pear juice containing PAT (0.08 or 0.4 μg/mL) after addition of the low level of ascorbic acid (143 μmol/mL) regardless of the level of ferrous iron were gradually increased as incubation time became longer. In contrast, the degradation rates in pear juice containing PAT after addition of the high level of ascorbic acid (286 μmol/mL) regardless of the level of ferrous iron were similar (almost 100%) between pear juice samples after 5 and 24 h incubation (Figure 4). It suggests that most of PAT in pear juice was degraded by addition of the high level of ascorbic acid and either level of ferrous iron after 5 h incubation, resulting in saturated PAT degradation rates.

Similar to the results in the presence of only ascorbic acid (Figure 3), the increase in the level of ascorbic acid (143 μg/mL to 286 μg/mL) did not affect the PAT degradation rates in apple juice containing PAT in the presence of the same level of ferrous iron after the same incubation time (Figure 5). However, the addition of the high level of ascorbic acid into pear juice showed an increased trend of the PAT degradation rates after 5 h incubation in the presence of the same level of ferrous iron (Figure 4), which are similar results to those in the presence of only ascorbic acid (Figure 2) but are different from those in apple juice containing the same level of PAT. Again, this discrepancy might have come from the difference between the initial contents of ascorbic acid in pear juice and apple juice.

Ascorbic acid is a powerful antioxidant and is generally recognized as safe (GRAS) and a relatively inexpensive, beneficial nutrient (vitamin C) [19]. It is also a food additive for beverages, which is commonly used to improve nutritional values or product quality by inhibition of browning reactions due to oxidation of polyphenols in the food industry [23]. It was documented that it does not negatively affect the sensory properties of apple juice [19]. Therefore, the use of ascorbic acid for PAT degradation is one of the promising methods for controlling the hazard posed by PAT in apple juice or pear juice. Since the PAT degradation rate leveled off after complete oxidation of ascorbic acid, the initial levels of ascorbic acid and ferrous ion as a catalyst for oxidation of ascorbic acid are important factors in PAT degradation [15]. In addition, iron from ferrous sulfate was used to prepare fortified milk for controlling iron-deficiency anemia in Brazilian children [35,36]. According to the dietary reference intakes for Korean adults, the recommended intake (RI) and tolerable upper intake level (UL) for vitamin C are 100 mg/day and 2000 mg/day, whereas those for iron are 10 mg/day and 45 mg/day, respectively [37]. In this study the ascorbic acid or ferrous iron concentrations (either 143 or 286 μmol/mL and either 0.033 or 0.11 μmol/mL, respectively) chosen for spiking the pear juice or apple juice were far below RI and UL for both vitamin C and iron. In conclusion, the use of ascorbic acid and ferrous sulfate in apple juice or pear juice contaminated with PAT could be of value for practical applications in food industries. This strategy could decrease the level of PAT in fruit juice to less than a detectable level.

## 4. Materials and Methods

### 4.1. Chemicals and Reagents

PAT standard (≥98.0% purity), HPLC grade acetic acid solution, and L-ascorbic acid (≥99.0%, crystaline) were purchased from Sigma-Aldrich Co. (St. Louis, MO, USA). Ethyl acetate was obtained from Daejung chemical Co. (Seoul, Korea). Anhydrous Sodium sulfate (Na_2_SO_4_) and ferrous sulfate (FeSO_4_·7H_2_O) were purchased from Junsei chemical Co. (Tokyo, Japan). Sodium carbonate (Na_2_CO_3_) was obtained from Samchun chemical Co. (Seoul, Korea). Acetonitrile (HPLC grade) was obtained from Supelco (Darmstadt, Germany). Pear juice and apple juice (100% pure) were purchased from Mippeum Living Healthy Co. (Busan, Korea) and Sandeuljeong Co. (Andong, Kyeongbuk, Korea), respectively.

### 4.2. Standard Solutions

PAT stock solution (200 μg/mL) was prepared by mixing 5 mg of PAT powder with 25 mL of ethyl acetate and stored at −20 °C until use. A series of PAT standard solutions (0.1, 0.2, 0.5, 1.0, and 2.0 μg/mL) were prepared freshly by dilutions of the stock solution with ethyl acetate. Then, 1 mL of each standard solution was evaporated to dryness under a gentle steam of nitrogen at 60 °C. The residue was dissolved in 1 mL of acidified water, which was adjusted to pH 4.0 with acetic acid.

### 4.3. Degradation of PAT in Pear Juice or Apple Juice Spiked with PAT Standard Solutions Together with Addition of Either Ascorbic Acid or Both Ascorbic Acid and Ferrous Iron, and Extraction of PAT in the Juice Samples

Pear juice or apple juice was spiked with PAT standard solutions to give concentrations of 0.08 μg/mL and 0.4 μg/mL in the spiked samples. Then, either ascorbic acid or ascorbic acid plus ferrous sulfate as ferrous iron were added to the pear juice or apple juice to give concentrations of 143 μg/mL or 286 μg/mL for ascorbic acid and 0.033 μmol/mL or 0.11 μmol/mL for ferrous iron, and the mixture was incubated at 25 °C for 5 or 24 h. After incubation, PAT was extracted from the mixture by the AOAC official method 995.10 with some modifications [38]. Briefly, each sample was extracted twice with ethyl acetate and washed with 1.5% sodium carbonate solution. Then, it was dried over anhydrous sodium sulfate and evaporated to dryness under nitrogen at 60 °C. The residue was dissolved in 1 mL of acidified water as described in preparation of the above standard solution and filtered through a 0.22 μm syringe filter (Pall US life science Co. Ann Arbor, MI, USA) prior to injection onto an HPLC. We calculated PAT degradation rates based on the recovery rates of PAT added into pear juice and apple juice (0% PAT degradation rate where 75 and 95% recovery rates for 0.08 μg/mL of PAT added into pear juice and apple juice, respectively, and 0% PAT degradation rate where 80 and 86% recovery rates for 0.4 μg/mL of PAT added into pear juice and apple juice, respectively).

### 4.4. HPLC Analysis

PAT was detected and quantified using an HPLC system (LC-20AD, Shimadzu; Tokyo, Japan) equipped with UVD (SPD-20A, Shimadzu; Tokyo, Japan). The determination of PAT was performed at 276 nm. Separation was carried out on a ZORBAX Eclips plus C18 column (4.6 mm × 250 mm, 5 μm particle size, Agilent; Santa Clara, CA, USA) and the column oven temperature was set at 25 °C. The mobile phase, 10% acetonitrile (ACN: DW = 10:90, *v*/*v*), was pumped at a flow rate of 0.5 mL/min, giving a total run time of 30 min. The injection volume of the samples was 100 μL.

### 4.5. Assessment of the Linearity, Precision, and Sensitivity of the Analytical Method for Determination of Levels of PAT

The linearity of a series of concentrations of PAT was assessed by a standard curve using five levels of PAT standard solutions (0.1, 0.2, 0.5, 1, and 2 μg/mL). The calibration curve of PAT was constructed by plotting the peak areas (y axis) versus PAT concentrations (x axis) in HPLC-UVD analyses. In order to determine the recovery rate of PAT, pear juice or apple juice was spiked with PAT standard solutions to give concentrations of 0.08, 0.2, and 0.4 μg/mL. PAT extraction from the spiked samples was performed by the procedures described above. After levels of PAT in the samples were analyzed by HPLC-UVD, they were expressed as mean ± standard deviation (SD).

The repeatability (within-day precision) was determined by three consecutive injections of patulin solutions extracted from the spiked samples within a day. The within-day precision was expressed as *RSDr* of PAT levels obtained in triplicate.

The sensitivity of the analytical method using HPLC-UVD was determined by LOD and LOQ. They were calculated by the following equation using the slope (S) of the calibration curve, which was obtained from linearity assessment, and the SD of the response: LOD = 3.3 × SD/S, LOQ = 10 × SD/S.

### 4.6. LC/MS/MS Analysis

LC/MS/MS was used to confirm the identity of PAT detected in pear juice and apple juice samples. The LC/MS/MS analysis was performed using an Agilent 1290 Infinity UHPLC (Santa Clara, CA, USA), which was coupled to an Agilent 6550 iFunnel Quadrupole Time-of-Flight (Q-TOF) LC/MS equipped with a dual-spray Agilent Jet Stream electrospray ionization source. Separation was carried out on an Agilent Poroshell 120 EC-C18 column (2.1 mm × 100 mm, 2.7 μm particle size) with a mobile phase at a flow rate of 0.4 mL/min. One mobile phase (A solution) consisted of 0.1% formic acid containing 5 mM NH_4_ formate, while another mobile phase (B solution) consisted of 0.1% formic acid in methanol containing 5 mM NH_4_ formate. A gradient elution program was applied as follows: after B solution was held on 10% from 0 min to 0.5 min, it was linearly increased from 10% at 0.5 min to 98% at 10 min and then held on 98% from 10 min to 15 min. Subsequently, 98% of B solution was linearly decreased from 98% at 15 min to 10% at 15.1 min and held on for 1.9 min for re-equilibration of the column before injection of the next sample. The injection volume of samples was 2 μL. The column temperature was maintained at 30 °C.

The mass spectrometer was operated in the positive ESI (electrospray ionization) mode, and the peak spectrum was obtained by the Find by Formula data-mining algorithm. The main MS parameters were optimized and set as follows: mass range, 40 to 1300 amu; scan rate, 3 spectra/s; collision energy, 10 V; Data processing was carried out using Agilent MassHinter Data Acquisition Software, rev. B.05.01 (Santa Clara, CA, USA).

### 4.7. Statistical Analyses

Data were statistically analyzed by t-test or a one-way analysis of variance (ANOVA) and expressed as the mean ± standard deviation using SigmaStat software (Jandel Corporation, San Rafael, CA, USA). A *p* value < 0.05 was considered statistically different.

## Data Availability

Not applicable.

## Supplementary Materials

The following supporting information can be downloaded at: https://www.mdpi.com/article/10.3390/toxins14110737/s1, Figure S1. Calibration curve of PAT standard solutions for quantification of PAT in (A) pear juice and (B) apple juice. A series of PAT standard solutions were prepared in the range of 0.1, 0.2, 0.5, 1, and 2 μg/mL. Each standard solution was injected into HPLC-UVD in triplicate; Table S1. Recovery rates and within-day precision of patulin in pear juice and apple juice.
