# Supplementary material for: Degradation of Patulin in Pear Juice and Apple Juice by Ascorbic Acid and the Combination of Ascorbic Acid and Ferrous Iron
Source: Toxins (Basel). 2022 Oct 28;14(11):737. doi: 10.3390/toxins14110737 (PMC9696537; doi:10.3390/toxins14110737)
Supplement: Supplementary file 1 [file toxins-14-00737-s001.zip › toxins-1965776 supplementary LAYOUT replaced.pdf]

toxins

A.

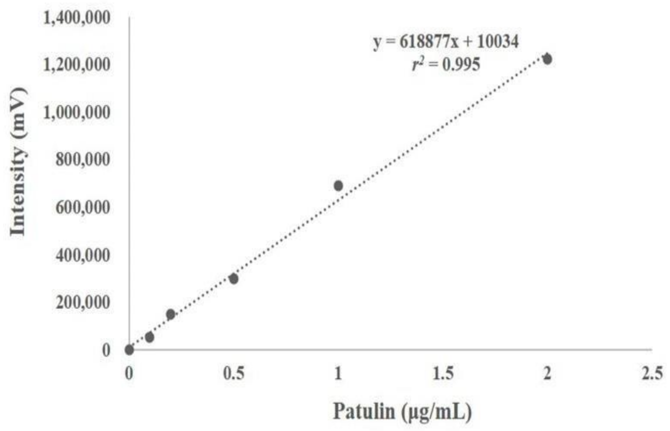

B.

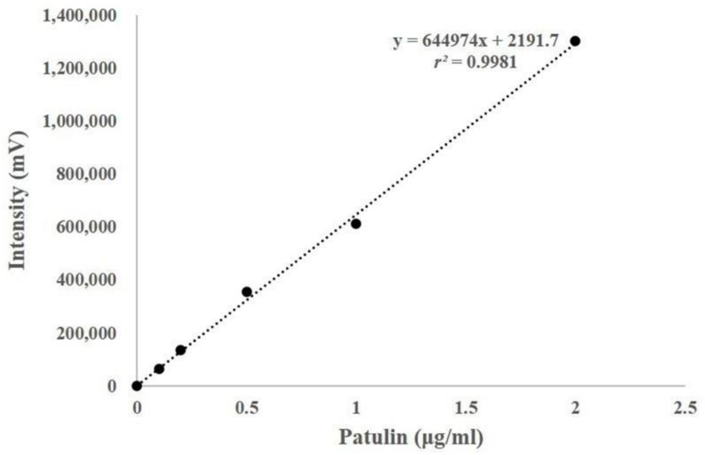

Figure S1. Calibration curve of PAT standard solutions for quantification of PAT in (A) pear juice and (B) apple juice. A series of PAT standard solutions wereprepared in the range of 0.1, 0.2, 0.5, 1, and 2 µg/ml. Each standard solution was injected into HPLC-UVD in triplicate.

Table S1. Recovery rates and within-day precision of patulin in pear juice and apple juice.

**Sample**	**Spiking Level (μg/ml)**	**Recovery Rate (%)**	***RSDr* * (%)**
Pear juice	0.2	75	7.5
	0.5	80	8.5
	1.0	81	8.2
Apple juice	0.2	95	6.3
	0.5	86	13.1
	1.0	86	12.7

* *RSDr* indicates relative standard deviation calculated from results generated under repeatability conditions.
